# Evaluating the Accuracy of Android Applications in Monitoring Environmental Noise Levels

**DOI:** 10.7759/cureus.81471

**Published:** 2025-03-30

**Authors:** Hamad Khan, Callum Findlay, Ruth-Ann Stevenson, T Singh

**Affiliations:** 1 Otolaryngology, Walsall Manor Hospital, Walsall, GBR; 2 Otolaryngology - Head and Neck Surgery, Southampton General Hospital, University Hospital Southampton NHS Foundation Trust, Southampton, GBR

**Keywords:** android applications, environmental noise monitoring, hearing protection, occupational health, smart phone accuracy

## Abstract

Objective

This study aimed to evaluate the accuracy of Android applications (apps) in measuring environmental noise levels, focusing on their potential use for occupational health assessments.

Methods

The top 10, highly rated, free Android apps were tested on a Samsung Galaxy A54 Smartphone (Samsung Group, Samsung Town, Seoul, South Korea) using pure tones at 100 Hz, 2000 Hz, and 4000 Hz, across four noise levels (25 dB, 40 dB, and 85 dB). Measurements were compared with a calibrated Precision Gold N09AQ environment meter (Maplin Electronics Ltd, Wath-Upon-Dearne, United Kingdom) in a controlled room. Data were analyzed using linear regression to determine R^2 ^values for each app.

Results

The control meter showed the highest accuracy (R^2 ^= 0.99). SPL Meter dB and sound Meter (KTW apps, Petaling Jaya, Selangor, Malaysia) and Sound Meter (Pony AI Inc.,Guangzhou, Guangdong, China) had the best performance (R^2 ^= 0.98). Accuracy declined at higher noise levels, with Sound Meter (ABC Apps) showing the least accuracy (R^2 ^= 0.85). User ratings did not correlate consistently with app performance.

Conclusion

Android apps offer potential as affordable noise measurement tools, with some apps demonstrating high accuracy at lower decibels. However, limitations such as reduced accuracy at higher decibels and lack of A-weighting for regulatory compliance hinder their use in professional settings. Further development and real-world testing are needed.

## Introduction

The issue of hearing loss is escalating in developed nations and has become one of the most prevalent work-related injuries [1]. In 2005, the United Kingdom (UK) government implemented the Control of Noise at Work Regulations, mandating the surveillance of all workplace environments in Great Britain to ensure that workers were not exposed to potentially harmful noise levels [2]. Similar occupational health guidelines have been outlined by the National Institute for Occupational Safety and Health in the United States [3,4]. 

The UK legislation was framed under the Health and Safety at Work Act of 1974 and aligns with the directive 2003/10/EC of the European Council [5]. This legislation requires employers to provide information and training to employees if personal noise exposure levels, on a daily or weekly basis, exceed 80 dB, and hearing protection is required if average exposure surpasses 85 dB (A-weighted) [5]. These guidelines and legislation provide a framework and legal obligation for employers to protect employees’ hearing. However, they are poorly enforced and complied with [2].

The Labour Force Survey (LFS) estimates that around 11,000 cases of noise-induced hearing loss were caused or worsened over the three-year period between 2020 to 2023 [6]. Legislation places the burden of responsibility on the employer and, therefore, requires the employer to identify where noise exposure of workers exceeds 85 dB and provide relevant hearing protection. Dedicated sound meters can be purchased for this purpose; however, these can be costly and require maintenance, servicing, and regular calibration, particularly for small businesses [7]. These factors potentially pose a barrier to employers complying with the legislation and protecting workers' hearing.

Literature has demonstrated that the technology available in modern smartphones and software applications (apps), may present an accurate and convenient substitute for professional sound meters [8,9]. Numerous studies have explored the use of iPhone (Apple Inc., Cupertino, California, United States) apps for assessing environmental noise levels using white noise to replicate broader environmental noise exposure [10,11]. Similarly, other studies evaluating the top-performing apps available on IOS and Android platforms demonstrated dependable accuracy compared to professional devices [8-10,12-14].

Given the widespread use of Android devices globally, this study aims to assess the accuracy of similar apps available on the Android operating system (Open Handset Alliance, Mountain View, California, United States), using pure tones. Pure tones allow for testing at specific frequencies relevant to occupational health, particularly in environments where certain machinery or equipment might produce sounds within these ranges [15].

## Materials and methods

Study design

A Samsung Galaxy A54 (Samsung Group, Samsung Town, Seoul, South Korea), Android version 13, software version 5.15.79, was used throughout the study. The phone was used without a protective case to optimise microphone accuracy. This model of phone represents an affordable and widely used smartphone, making it a practical choice for small businesses. The Google Play Store (Google LLC, Mountain View, California, United States) was searched in April 2023 using the terms “noise” and “decibel” and the 10 highest-rated free Android applications (rated ≥4.5 stars) were selected. No advertisements or add-ons were purchased. The majority of the apps had no in-app calibration option; therefore, no calibration was used to reflect typical consumer use.

Study setting and equipment

Testing was completed over two days, on June 11-12, 2023, in a sound-attenuated room at the Royal South Hants Hospital, Southampton, UK. This environment minimized external noise interference. Pure tone stimuli (100 Hz, 2000 Hz, and 4000 Hz) were generated using a calibrated speaker to simulate noise ranges common in occupational environments. The audiogram assessment adhered to the industry-standard protocol involving a three-frequency evaluation [15].

App testing procedure

Noise levels of 25 dB, 40 dB, 55 dB, and 85 dB were played through the speaker. The smartphone was positioned 1 meter from the sound source and facing directly (0^o^ azimuth) toward the speaker. Measurements were taken at five-second intervals, and mean values were calculated. Test-retest reliability was evaluated by conducting trials at different times of the day.

Control and reliability

A Precision Gold N09AQ Meter (Maplin Electronics Ltd, Wath-Upon-Dearne, UK) was used as a control. The device was calibrated before the study to ensure accuracy. Control measurements followed the same protocol as app testing.

Statistical analysis

The data analysis was performed using Microsoft Excel (Version 2305 Build 16.0.16501.20074) 64-bit (Microsoft Corporation, Redmond, Washington, United States). Linear regression analysis was applied to the dataset, and for each individual application, goodness-of-fit values R^2^ were computed. The R^2^ value serves as an indicator of how effectively the app predicts the actual noise thresholds detected during the study. A higher R^2^ value signifies a greater predictive accuracy, with an R^2^ value approaching 1 indicating a strong predictive capability of the app in estimating the true noise thresholds.

## Results

The Precision Gold N09AQ Environment Meter used as control had the highest accuracy, with an R^2^ value of 0.99, and demonstrated reliability when measuring high noise levels. Of the 10 apps tested, SPL Meter dB and Sound Meter (KTW apps, Petaling Jaya, Selangor, Malaysia) demonstrated the highest accuracy (R^2^ = 0.98), followed by Sound Meter (Pony AI Inc., Guangzhou, Guangdong, China), and Noise Detector & Decibel Meter (Yalintech, Amsterdam, Netherlands), both with R^2^ = 0.98 (Table 1). Three measurements for each testing noise level were taken at five-second intervals, and their mean values were recorded.

**Table 1:** Mean noise level readings and goodness-of-fit evaluation. Mean values were calculated for each frequency and decibel using an average of all three readings.

	App	25dB	40 dB	55 dB	85 dB	Goodness of fit
Control	Precision Gold N09AQ Environment Meter (Maplin Electronics Ltd, Wath-Upon-Dearne, United Kingdom)	28.8 ± 2.0	41.6 ± 2.53	56.1 ± 2.9	85.3 ± 1.9	0.99
First	SPL Meter: DB & Sound meter (KTW apps, Petaling Jaya, Selangor, Malaysia)	26.4 ± 0.7	33.0 ± 1.4	47.0 ± 1.2	76.0 ± 1.1	0.98
Second	Sound Meter (Pony AI Inc.,Guangzhou, Guangdong, China)	28.2 ± 0.6	35.2 ± 2.7	48.3 ± 4.5	78.5 ± 2.1	0.98
Third	Noise detector & Decibel meter (Yalintech, Amsterdam, Netherlands)	29.2 ± 5.0	35.4 ± 2.4	50.3 ± 3.1	80.0 ± 0.3	0.98
Fourth	Sound Meter (Splend Apps, Wrocław, Poland)	28.5 ± 0.7	35.5 ± 1.9	49.0 ± 3.6	79.5 ± 1.2	0.98
Fifth	Sound Meter - Decibel (melon soft)	20.0 ± 2.3	27.2 ± 3.1	41.0 ± 3.8	74.0 ± 1.8	0.97
Sixth	Sound Meter (KUCO Apps)	29.3 ± 0.5	36.5 ± 1.7	51.0 ± 1.5	86.2 ± 3.3	0.97
Seventh	Sound Meter (coolexp, Hanoi, Vietnam)	30.5 ± 1.0	36.3 ± 1.0	50.0 ± 2.7	80.1 ± 1.6	0.97
Eighth	Decibel Meter (EXA Tools, Bielsko-Biała. Poland)	26.0 ± 1.2	32.1 ± 2.4	45.1 ± 6.3	52.1 ± 39.1	0.93
Nineth	Sound Meter HQ PRO (Just4Fun Utilities, Pruszkow, Poland)	28.0 ± 0.7	30.0 ± 1.0	36.1 ± 3.2	65.0 ± 4.2	0.91
Tenth	Sound meter (ABC apps)	14.1 ± 10.6	27.0 ± 5.5	45.0 ± 4.1	49.3 ± 37	0.85

**Figure 1:**
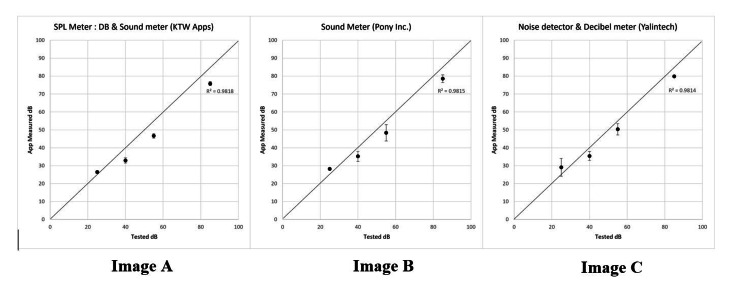
Comparison of app-measured dB vs. tested dB for the top three noise measurement apps, with R² values displayed. Image A: Comparison of app measured dB versus tested dB for SPL meter: dB & sound meter (KTW app). Image B: Comparison of app measured dB versus tested dB for Sound meter (Pony inc). Image C: Comparison of app measured dB versus tested dB for Noise detector & Decibel meter (Yalintech) dB: decibel

The least accurate app, Sound Meter (ABC Apps), had an R2 of 0.85 and consistently underestimated noise levels, particularly at higher decibel thresholds, such as 85 dB. While nine out of 10 apps achieved R2 >0.9 at lower decibel levels (e.g., 25-40 dB), performance declined notably at 55 dB and 85 dB (Table 2, Figure 1). Figure 1 highlights the performance variance across apps, with error bars representing standard deviations.

The Google Play Store ratings for the apps ranged from 4.5 to 4.8, while the number of user ratings varied widely, from 1146 to 324,136 (Table 2). Despite this, app popularity (reflected in ratings) did not consistently correlate with performance. For example, Sound Meter by Splend Apps (Wrocław, Poland) had the highest user ratings (324,136) and a 4.8-star rating but did not outperform less-rated apps such as SPL Meter: dB & Sound Meter in terms of accuracy.

**Table 2:** Summary statistics for all android apps evaluated, in order of star rating

Android App	Star Rating	Number of Ratings	Number of Languages	Manufacturer	Original Year on App Store	A-Weighting
Sound Meter	4.8	32,4163	1	Splend Apps, Wrocław, Poland	2014	Not Specified
Sound Meter	4.8	8406	1	Cool Experience Apps (coolexp), Hanoi, Vietnam	2022	Not Specified
Sound Meter HQ PRO	4.6	7239	1	Just4Fun Utilities, Pruszkow, Poland	2020	Not Specified
Sound Meter	4.5	17,1533	1	Abc Apps, Unknown	2015	Not Specified
SPL Meter: dB & Sound Meter	4.6	25266	1	KTW apps, Petaling Jaya, Selangor	2018	Not Specified
Noise Detector 7 Decibel Meter	4.5	1423	13	Yalintech, Amsterdam, Netherlands	2020	Not Specified
Sound Meter	4.5	1146	1	KUCO Apps, Unknown	2022	Not Specified
Decibel Meter	4.5	6354	1	Exa Tools, Bielsko-Biala, Poland	2016	Not Specified
Sound Meter	4.8	26,516	56	Pony Inc., Guangzhou, Guangdong, China	2021	Not Specified
Sound Meter-Decibel	4.7	94,923	1	Melon Soft, Unknown	2016	Not Specified

Most apps offered only single-language interfaces, limiting accessibility for a global audience. Only two apps (Noise Detector 7 Decibel Meter by Yalintech and Sound Meter by Pony Inc.) offered multiple language selections. Of the two, Sound Meter (Pony Inc.) offered an impressive array of 56 different languages. The original year on the App Store ranged between 2014 and 2022. None of the applications featured an A-weighting option.

## Discussion

This study assesses the feasibility and accuracy of using Android apps using a mid-range Samsung smartphone to measure noise exposure levels and highlights critical limitations. Our findings show that nine out of 10 apps achieved a high R^2^ value greater than 0.9. However, a significant decrease in accuracy was noted at higher sound levels (e.g., at 55 dB and 85 dB), with variations of up to 10 dB or more in some cases. Rabinowitz et al. emphasized the importance of small variations in the accuracy, which can significantly underrepresent occupational risks, particularly where protective devices are required [17,18]. This demonstrates the importance of the careful selection of apps and possible in-app feature enhancements for possible improvement in professional environments.

The lack of in-app calibrations, particularly the absence of A-weighting, is a crucial limitation. A-weighting is required for compliance with regulations such as the Control of Noise at Work Regulations (2005), as it adjusts sound measurement to reflect human hearing sensitivity. Without A-weighing, a highly accurate app could lack professional applicability, leading to questionable reliability in an occupational health context [5,16]. Notably, the variability in app performance poses another challenge to relying on consumer-focused tools for accurate measurements. While apps such as Sound Meter HQ Pro (Just4Fun Utilities) and Sound Meter (ABC Apps) had high R^2^ values of 0.91 and 0.85, respectively, their significant underestimation of higher decibels limits their utility in professional settings.

A methodology was adopted solely to evaluate apps with the highest ratings in each price bracket, using customer reviews as an indicator of the most optimal app [16]. Notably, in our study, SPL Meter:dB and Sound Meter achieved the highest accuracy (4.6 stars), and apps with higher user ratings (e.g., 324,143) were not necessarily more precise. Interestingly, the top-rated app ranked fourth in R^2^ performance, revealing a disconnect between popularity and accuracy.

Earlier studies favoured iOS apps as being more reliable for sound level measurements [9,18]. However, the strong performance of some of the apps in our study demonstrating high R^2^ values suggests that Android apps are increasingly closing the gap. Direct comparisons between Android and iOS under identical testing conditions could clarify their use in real work settings.

This study also highlights challenges related to usability and accessibility, as the majority of the apps offered only single-language interfaces, limiting accessibility. Only one app provided extensive language options, highlighting concerns for global accessibility, particularly in developing countries [17]. Additionally, this study used controlled conditions to ensure consistency, and such settings may not fully represent real-world scenarios. Research has shown that factors such as acoustic reflections, background noise, and environmental variability can significantly alter noise measurements [12,19]. Future studies should evaluate app performance in dynamic environments to assess practical usage.

Limitations and future directions

This study only evaluated free apps to reflect the most accessible user option. However, paid versions with calibration may offer superior accuracy, particularly at higher frequencies, as demonstrated in previous research [8,11]. Exploring the use of paid apps in future research, alongside the use of calibration in dynamic settings, could further enhance Android app performance, making them more useful for professional and regulatory use.

## Conclusions

This study demonstrates the potential of Android apps as cost-effective tools for noise monitoring, with certain apps showing high accuracy at lower decibels under controlled settings. However, the lack of accuracy at higher decibels, absence A-weighting for regulatory compliance, and limited usability features restrict their use in professional scenarios. This implies that in an occupational health setting, professional sound meters should be used to measure the exposure to noise, as over-reliance on current Android apps could have detrimental effects on health. 

Further studies are required to address these limitations through enhanced accuracy at elevated noise levels, integrating calibrated options and validating performance in real-world environments, these apps could become reliable and practical alternatives to traditional sound level meters, particularly in resource limited or remote settings. 
